# ObsPlus: A Pandas-centric ObsPy expansion pack

**DOI:** 10.21105/joss.02696

**Published:** 2021-04-15

**Authors:** Derrick J.A. Chambers, M. Shawn Boltz, Calum J. Chamberlain

**Affiliations:** 1 National Institute for Occupational Safety and Health, Spokane Mining Research Division; 2 School of Geography, Environment and Earth Sciences, Victoria University of Wellington, New Zealand

## Abstract

Over the past decade, ObsPy, a python framework for seismology (Krischer et al., 2015), has become an integral part of many seismology research workflows. ObsPy provides parsers for most seismological data formats, clients for accessing data-centers, common signal processing routines, and event, station, and waveform data models.

ObsPlus significantly expands ObsPy’s functionality by providing simple data management abstractions and conversions between ObsPy classes and the ubiquitous pandas DataFrame (McKinney, 2010).

## Statement of Need

ObsPlus benefits researchers by 1) simplifying seismological data access patterns and management of local data, 2) providing means to extract facets of seismic catalog hierarchies to tabular forms, and 3) enabling the packaging and distribution of complete seismological datasets.

## Functionality and Features

1. **A data retrieval interface** ObsPlus provides a unified data retrieval interface for in-memory, on disk, and remote seismological data. This is enabled by in-process databases which provide a simple mechanism to index and access local seismological data stored in directories of arbitrary organization. Importantly, the classes implement a superset of the interface already provided by ObsPy’s remote clients, making it straight-forward to write data-source agnostic code.
2. **Alternative data structures** While ObsPy’s data structures are quite powerful, they are not always the most convenient. For example, the canonical event data representation is a deeply nested tree-like structure based on the QuakeML standard (Schorlemmer et al., 2011). Working with events in ObsPy often necessitates deeply nested recursive code which can become difficult to understand and maintain. ObsPlus provides functionality to flatten desired components of these tree structures into DataFrames which are simpler and more efficient when the full complexity of QuakeML isn’t merrited for the task at hand.
3. **Datasets** ObsPlus provides a simple mechanism to bundle, distribute, download, and interact with complete seismological datasets. This is done by creating a simple python package (for which we provide a cookie cutter template) which is published to PyPI. Each package includes small files and instructions to download large files. Optionally, a list of files and their corresponding hashes can be used to validate downloaded data. Datasets are then discovered and loaded through python’s plugin system, and downloaded when needed.
4. **Utilities** ObsPlus’s list of utilities is quite long and more are being added regularly. Many are focused around validating and manipulating event data.

ObsPlus has become an important part of the National Institute for Occupational Safety and Health (NIOSH)’s data processing and management workflows and has enabled rapid prototyping of new ideas while managing complexity through appropriate abstractions. It is our hope that ObsPlus will provide similar benefits to the broader seismology community.
